# Effects of acute ingestion of a pre-workout dietary supplement with and without *p-*synephrine on resting energy expenditure, cognitive function and exercise performance

**DOI:** 10.1186/s12970-016-0159-2

**Published:** 2017-01-12

**Authors:** Y. Peter Jung, Conrad P. Earnest, Majid Koozehchian, Elfego Galvan, Ryan Dalton, Dillon Walker, Christopher Rasmussen, Peter S. Murano, Mike Greenwood, Richard B. Kreider

**Affiliations:** 1 Exercise & Sport Nutrition Lab, Department of Health & Kinesiology, Texas A&M University, College Station, TX 77843-4243 USA; 2 Nutrabolt, Bryan, TX 77807 USA; 3 Center for Translational Research in Aging and Longevity, Department of Health & Kinesiology, Texas A&M University, College Station, TX 77843-4243 USA; 4 Institute for Obesity Research & Program Evaluation, Department of Nutrition and Food Sciences, Texas A&M University, College Station, TX 77843 USA

**Keywords:** Ergogenic Aids, Dietary Supplement, Energy Drinks

## Abstract

**Background:**

The purpose of this study was to examine the effects of acute ingestion of a pre-workout dietary supplement (PWS) with and without *p-*synephrine (S) on perceptions of readiness to perform, cognitive function, exercise performance, and markers of safety.

**Methods:**

In a randomized, double-blind, and counterbalanced manner; 25 healthy and recreationally active male and female participants ingested a flavored maltodextrin placebo (PLA), a PWS containing beta-alanine (3 g), creatine nitrate as a salt (2 g), arginine alpha-ketoglutarate (2 g), N-Acetyl-L-Tyrosine (300 mg), caffeine (284 mg), *Mucuna pruiriens* extract standardized for 15% L-Dopa (15 mg), Vitamin C as Ascorbic Acid (500 mg), niacin (60 mg), folate as folic acid (50 mg), and Vitamin B12 as Methylcobalamin (70 mg) with 2 g of maltodextrin and flavoring; or, the PWS with *Citrus aurantium* (PWS + S) extract standardized for 30% *p*-synephrine (20 mg). Participants had heart rate (HR), blood pressure, resting energy expenditure (REE), 12-lead electrocardiograms (ECG), perceptions about readiness to perform, cognitive function (Stroop Color-Word test), bench and leg press performance (2 sets of 10 repetitions at 70% of 1RM and 1 set to failure), and Wingate anaerobic capacity (WAC) sprint performance determined as well as donated blood samples prior to and/or following exercise/supplementation. Data were analyzed by MANOVA with repeated measures as well as mean changes from baseline with 95% confidence intervals (CI).

**Results:**

No clinically significant differences were observed among treatments in HR, blood pressure, ECG, or general clinical blood panels. There was evidence that PWS and PWS + S ingestion promoted greater changes in REE responses. Participants reported higher perception of optimism about performance and vigor and energy with PWS and PWS + S ingestion and there was evidence that PWS and PWS + S improved changes in cognitive function scores from baseline to a greater degree than PLA after 1 or 2 h. However, the scores in the PWS + S treatment did not exceed PLA or PWS responses at any data point. No statistically significant differences were observed among treatments in total bench press lifting volume, leg press lifting volume or WAC sprint performance.

**Conclusions:**

Within the confines of this study, ingestion of PWS and/or PWS + S prior to exercise appears to be well-tolerated when consumed by young, healthy individuals. The primary effects appear to be to increase REE responses and improve perceptions about readiness to perform and cognitive function with limited to no effects on muscular endurance and WAC. The addition of 20 mg of *p*-synephrine to the PWS provided limited to no additive benefits.

**Trial registration:**

This trial (NCT02952014) was retrospectively registered on September 13th 2016.

## Background

Research has shown that ingestion of some ergogenic nutrients and/or caffeinated beverages prior to exercise can improve mental focus and/or exercise capacity [1, 2]. For this reason, a number of energy drinks and pre-workout supplements (PWS) have been developed and marketed to active individuals. The primary ergogenic properties in most of these supplements appears to be water, carbohydrate, and caffeine [1]. However, more recently PWS’s have been developed that not only contain nutrients that may affect acute exercise performance (e.g., carbohydrate, caffeine, nitrates, etc.) but also nutrients that can increase energy expenditure, reduce catabolism, and/or promote protein synthesis (e.g., amino acids, creatine, β-alanine, etc.) [1–3]. Theoretically, use of PWS’s prior to exercise may enhance mental focus, cognitive function, and/or exercise capacity and regular use of PWS’s during training may lead to greater training adaptations. Consequently, there has been increased interest in examining the acute and chronic safety and efficacy of PWS’s marketed to active individuals as well as whether adding potentially ergogenic nutrients to PWS’s may promote additive benefits [1].

This study examined the safety and efficacy of acute ingestion of a market leading PWS on ratings of perception of readiness to perform, resting energy expenditure and metabolism, cognitive function, exercise performance, and markers of safety. The PWS studied contained several nutrients at previously reported effective doses that have ergogenic properties including caffeine [4], beta-alanine [5], creatine [6], nitrate [7, 8], arginine alpha-ketoglutarate [9] as well as other nutrients purported to effect cognitive function like tyrosine [10, 11] and *Mucuna pruriens* containing L-Dopa [12, 13]. It is well established that consuming caffeine prior to exercise (e.g., 3–6 mg/kg) can improve exercise performance, cognitive function, and vigilance [1, 4]. A number of studies also indicate that ingestion of nitrate prior to exercise (e.g., 300 mg) can improve exercise capacity [7, 8, 14–17]. Theoretically, ingesting these nutrients at effective doses in PWS’s prior to exercise may improve perceptions of readiness to perform, cognitive function, and/or exercise performance leading to higher quality workouts. If so, regular use of these types of PWS may effect quality of training and/or training adaptations particularly if they contain nutrients that have been reported to enhance training adaptations like beta-alanine [5, 18–24] and/or creatine [6, 25].

*Citrus aurantium* is found in the peel of bitter orange and contains *p*-synephrine which is a protoalkaloid with sympathomimetic properties [26–28]. *Citrus aurantium* (generally containing 20–100 mg of *p*-synephrine) has been purported to serve as a mild central nervous system stimulant [26, 28], suppress appetite [29], increase resting energy expenditure and affect carbohydrate and fat oxidation rates [30–33], and promote weight loss [34–36] with no negative effects on the cardiovascular system [37–39]. There is also evidence that *Citrus aurantium* ingestion can effect memory [40, 41] and resistance-exercise performance [30]. Theoretically, adding *Citrus aurantium* to a PWS may promote greater resting energy expenditure, cognitive function, and/or exercise capacity during an exercise bout. The purpose of this study was to examine the safety and efficacy of ingesting a market leading PWS with and without *p*-synephrine on ratings of perception of readiness to perform, cognitive function, resting energy expenditure and metabolism, exercise performance, and markers of safety. This paper presents results from a study evaluating the acute effects of PWS ingestion with and without *p*-synephrine while the effects of ingesting these PWS on training adaptations are presented in a companion paper.

## Methods

### Research design

This study was conducted in a randomized, double blind, counter-balanced, and crossover manner. Subjects participated in a familiarization session and three treatment testing sessions with a 1-week washout period observed between each testing session. The study was conducted at the Exercise & Sport Nutrition Laboratory (ESNL) at Texas A&M University after obtaining approval from the university ethics committee. The following description of methods and procedures provides an overview of the study.

### Participant recruitment and familiarization

Participants were recruited to participate in this study from local advertisements. Inclusion criteria required that each participant have at least 6 months of resistance training experience immediately prior to entering the study inclusive of performing bench press and leg press or squat exercises. Participants were excluded if they noted a history of treatment for metabolic disease, hypertension, thyroid disease, arrhythmias, cardiovascular disease; and/or, if they were currently using any prescription medication. Further exclusion criteria also included an intolerance to caffeine and/or other natural stimulants; pregnant or lactating women; a history of smoking; and, excessive alcohol consumption (>12 drinks/wk).

**Fig. 1:**
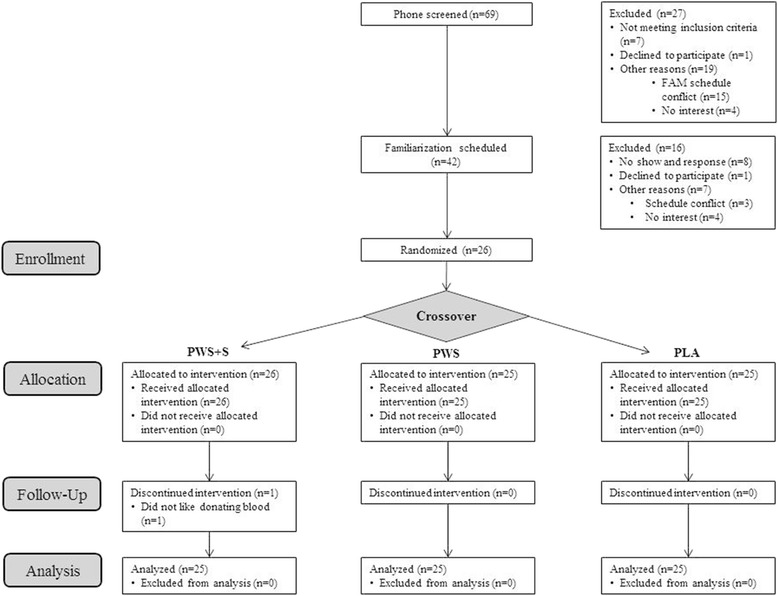
CONSORT schematic of enrollment and treatment allocation to the study

Figure 1 presents a CONSORT schematic of enrollment and treatment allocation to the study. A total of 69 individuals responded to advertisements. Of these, a total of 42 individuals met initial study entry criteria via phone interview and were invited to a familiarization session where the details of the study were explained, informed consent was obtained in compliance with our Institutional Review Board, medical history was assessed, and a general medical exam was performed. Height, weight, and body composition was determined. Participants then had one repetition maximum (1 RM) bench press and 1RM leg press determined. Participants also practiced the anaerobic sprint test used in the study. A total of 26 individuals began the study. One participant withdrew due to not feeling comfortable with donating blood samples after the first testing session. A total of 25 individuals participated in the study.

### Testing sequence

**Fig. 2:**
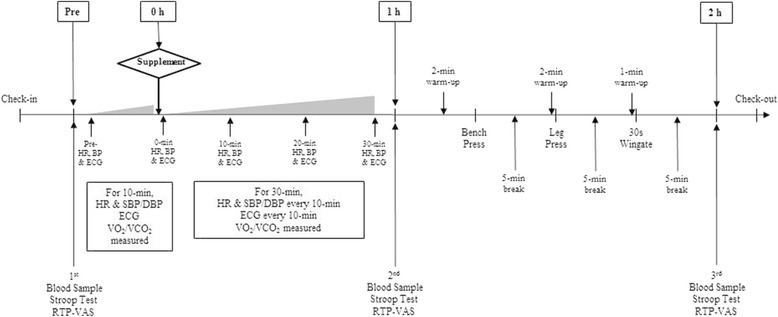
Study timeline

Figure 2 shows the timeline of testing procedures. Prior to each testing session, participants were instructed to refrain from exercise, caffeine, and supplements and/or medications containing stimulants for 48 h prior to testing. Participants were asked to present to the lab after a 12 h fast. Once arriving to the lab, participants donated ~ 20 ml of blood via venipuncture of an antecubital vein using standard procedures. Following blood sampling, participants completed a readiness to perform (RTP) visual analogue scale (VAS) and completed a Stroop Color-Word cognitive function test. Participants were then placed on an exam table in the supine position while electrodes were placed on the participant. The metabolic cart was then calibrated. After at least 10 min of rest in the supine position; resting heart rate, blood pressure, and 12 lead electrocardiographs (ECG) were obtained. Following this procedure, resting energy expenditure (REE) measurements were obtained for 10 min.

Participants were then randomly given in a counterbalanced and double-blinded manner either one serving (12 g) of a flavored maltodextrin placebo (PLA); a PWS containing β-alanine (3 g), creatine nitrate as a salt (2 g), arginine alpha-ketoglutarate (2 g), N-Acetyl-L-Tyrosine (300 mg), caffeine (284 mg), *Mucuna pruiriens* extract standardized for 15% L-Dopa (15 mg), Vitamin C as Ascorbic Acid (500 mg), niacin (60 mg), folate as folic acid (50 mg), and Vitamin B_12_ as Methylcobalamin (70 mg) with the remainder of the supplement consisting of flavored maltodextrin to equal 12 g (*Nutrabolt Inc., Brayan, TX*); or, (3) the PWS with *Citrus aurantium* (PWS + S) extract standardized for 30% *p*-synephrine (20 mg) (*Nutratech Inc., Caldwell, NJ*). Supplements were independently packaged by an independent third party into coded single foil packets for double-blind administration following Good Manufacturing Practices and certified to contain the aforementioned ingredients by VMI Nutrition (*Salt Lake City, UT*). The contents of the supplement packets were mixed into approximately 235 ml of water.

After the participant ingested the supplement; supine heart rate, blood pressure, and ECG’s were obtained every 10-min during a 30-min REE test. Following REE assessment and approximately 1 h after ingestion of the supplements, participants donated a post-supplementation / pre-exercise blood sample and had RTP-VAS and Stroop cognitive function determined. Participants then performed a 2 min warm up followed by performing 3 sets of 10 repetitions at 70% of 1 RM on the bench press interspersed by 2 min rest between sets. During the third set, participants were asked to complete as many repetitions as possible to failure. Participants then rested for 5 min, warmed up for 2 min, and then performed 2 sets of 10 repetitions and 1 set to failure at 70% of 1RM on the leg press following similar procedures. Following a 5 min recovery, participants then performed a 30 s WAC on a cycle ergometer. Participants then had post-exercise RTP-VAS and Stroop cognitive function determined and donated a final blood sample 2 h after ingestion of the supplements. The experiment was repeated using the alternate supplement administered in a counterbalanced manner two additional times following a 1 week washout after each additional testing session.

## Procedures

### Body composition

Body mass and height were determined according to standard procedures using a Healthometer Professional 500KL (*Pelstar LLC, Alsip, IL, USA*) self-calibrating digital scale with an accuracy of ± 0.02 kg. Whole body composition measures (excluding cranium) were determined with a Hologic Discovery W Dual-Energy X-ray Absorptiometer (DEXA; *Hologic Inc., Waltham, MA, USA*) equipped with APEX Software (*APEX Corporation Software, Pittsburg, PA, USA*) by using procedures previously described [42].

### Muscular strength and endurance

Bench press tests were performed using a standard isotonic Olympic bench press (*Nebula Fitness, Versailles, OH*) while leg press was determined using a hip/leg sled (*Nebula Fitness, Versailles, OH*) using standard procedures [43]. Bench press and leg press 1RM’s were determined during the familiarization session by having the participants follow a standard warm-up consisting of 10 repetitions using 50% of their estimated 1RM, 5 repetitions using 70% of their estimated 1RM, and 1 repetition using 90% of their estimated 1RM. Participants were given 2 min recovery between attempts and performed 1RM lifts until reaching a failure weight. After 5 min recovery, participants warmed-up in a similar fashion as described above and then performed 1RM lift attempts on a standard hip sled/leg press (*Nebula Fitness, Versailles, OH*) according to standard procedures [43]. For 1 RM determination, strong verbal encouragement was provided. Hand, seat, and foot placement positions were recorded to standardize positions among testing sessions. During the muscular endurance test, participants were asked to perform 2 sets of 10 repetitions at 70% of 1RM, if possible, with 2-min rest between sets. During the final set, participants were asked to complete as many repetitions as possible until failure. Total lifting volume was calculated by multiplying the amount of weight lifted times the number of successful repetitions completed during each set. Day to day test reliability of performing this endurance test in our lab on resistance-trained participants has yielded a standard error of measurement (SEM) of 92 kg, a SEM as a percent of grand mean of 4.1%, a CV of 0.34, and an intraclass correlation coefficient of 0.99 for 3 sets of bench press total lifting volume; and, a SEM of 820 kg, an SEM as a percent of grand mean of 6.4%, a CV of 0.32, and an intraclass correlation coefficient of 0.96 for 3 sets of leg press total lifting volume.

### Cardiovascular markers

Heart rate was determined by palpation of the radial artery while blood pressure was determined using standard auscultatory procedures [44]. Resting 12-lead ECG’s were obtained using a Nasiff Cardio Card electrocardiograph (*Nasiff Associates, Inc, Central Square, NY, USA*).

### Resting energy expenditure

REE assessments were conducted according to standard protocols using a metabolic cart (*Parvo Medics TrueMax 2400 Metabolic Measurement System, ParvoMedics, Inc, Sandy, UT, USA*). This test was conducted in a fasted state with the participants lying supine on an exam table. A clear metabolic canopy was placed over the participant’s head and neck, so that resting ventilation, oxygen uptake (VO_2_), and carbon dioxide (VCO_2_) measurements could be determined. The participants remained motionless without falling asleep for approximately 10 min before supplementation and then about 30 min after supplementation. Metabolic measurements were recorded every minute over the testing period.

### Readiness to perform assessment

Ratings of perceptions of readiness to perform were assessed using a visual analogue scale using a 5-item descriptive scale (strongly disagree, disagree, neutral, agree, strongly agree) arranged on a 20 cm dotted bar with these terms equidistant along the scale. Participants were asked to respond to the following statements: “*I am looking forward to today’s workout*”; “*I am optimistic about my future performance*”; “*I feel vigorous and energetic*”; and, “*I have little muscle soreness*” by circling the number or dot between numbers that best described their current perceptions related to these questions. Day to day test reliability of administering this test in our lab has yielded SEM’s of 0.35, 0.25, 0.28, 0.72; SEM as a percent of grand mean of 9.6%, 7.1%, 8.6%, 20.9%; CV’s of 0.23, 0.19, 0.26, and 0.34; and intraclass correlation coefficients of 0.83, 0.86, 0.89, and 0.63 for questions 1 through 4, respectively.

### Cognitive function assessment

Cognitive function was assessed using the Stroop Word-Color test [45, 46] which has widely-used and validated in a variety of populations [46–50]. The test consists of three separate pages / tests with 100 items, presented in 5 columns of 20 items. Items on the first page (Word) are the color words RED, GREEN, and BLUE in black ink. On the second page (Color) the items are XXX’s colored in red, green, or blue ink. Items on the third page (Word-Color) are the words RED, GREEN, and BLUE printed in red, green, or blue ink with the limitation that word and ink could not match. Participants were given standardized instructions and asked to read aloud each word or color on each page as fast as they could for 45-s on page/test. The number of correct responses obtained during the time period is used to assess cognitive function. Day to day test reliability of administering this test in our lab has yielded a SEM’s of 4.9, 9.1, and 10.4 counts; an SEM as a percent of grand mean of 4.3, 10.5 and 16.7%; CV’s of 0.14, 0.19, and 0.25; and, intraclass correlation coefficients of 0.90, 0.68, 0.57 for Word, Color and Word-Color, respectively.

### Anaerobic capacity test

Wingate anaerobic capacity sprint tests were performed on a Lode Excalibur Sport Ergometer (*Lode Excalibur Sport Ergometer, Lode BV, Groningen, Netherlands*) with work rate set at of 7.5 J•kg^−1^•rev^−1^. Participants were asked to pedal as fast as possible prior to application of the workload and sprint at an all-out maximal capacity for 30 s. Day to day variability in performing Wingate anaerobic capacity tests in our laboratory yielded an SEM of 53 W, an SEM as a percent of grand mean of 8.9%, a CV 0.26, and an intraclass correlation coefficient of 0.89 for mean power.

### Blood chemistry assessment

Plasma creatine was assayed prior to and after 1 and 2 h of supplementation as a general indicator of supplement bioavailability using calorimetric assay kits (*Sigma-Aldrich, St. Louis, MO*), Test-to-test variability of performing these assays yielded a SE of 0.09, an SEM as a percent of grand mean of 6%, mean CV of ± 0.1, and an intraclass correlation coefficient of 0.99. Serum alkaline phosphatase (ALP), aspartate transaminase (AST), alanine transaminase (ALT), creatine kinase (CK), lactate dehydrogenase (LDH), blood urea nitrogen (BUN), creatinine, total cholesterol (CHL), high density lipoprotein [HDL-c], low density lipoprotein [LDL-c], triglycerides [TG]), and glucose were assayed using a Cobas c 111 (*Roche Diagnostics, Basel, Switzerland*) prior to and following 2 h after supplementation. This analyzer has been known to be highly valid and reliable in previously published reports [51]. The internal quality control was performed using two levels of control fluids purchased from the manufacturer to calibrate to acceptable standard deviations (SD’s) and CV’s. Samples were re-run if the observed values were outside control values and/or clinical norms according to standard procedures. Test-to-test reliability (10-days) assessment yielded reliability CV’s ranging between 0.4 and 2.4% for low control samples and 0.6–1.9% for high controls with precision ranging between 0.8 and 2.4% on low and 0.5–1.7% for high controls.

### Statistical analysis

Data were analyzed using general linear models (GLM) multivariate analysis of variance (MANOVA) with Wilks’ Lambda and Greenhouse-Geisser adjustments using SPSS version 22.0 software (*IBM SPSS Statistics, Chicago, IL*). When a significant treatment, time and/or interaction alpha level was observed, Tukey’s least significant difference post-hoc analysis was performed to determine where significance was obtained. Area under the curve (AUC) was calculated on select variables using GraphPad Prism 6 software (*GraphPad Software, Inc., La Jolla, CA*). Delta values were calculated from subtracting baseline values with means and 95% Confidence Interval (CI) to determine whether changes from baseline and/or among treatments were significant [52]. Means were considered significantly different when the probability of error was 0.05 or less with statistical tendencies and effect size calculations noted when p-levels ranged between *p* > 0.05 to *p* < 0.10. All data are presented as mean ± SD unless otherwise noted.

## Results

### Participant demographics

Table 1 presents participant demographics. A total of 25 individuals completed the study (20 males and 5 females). Participants were 22 ± 3 y, 176.1 ± 8.2 cm, 78.2 ± 13.0 kg, 15.2 ± 5.2% fat, and 25.09 ± 3.0 kg/m^2^. No participant experienced an adverse medical event as result of participation in this study that required medical treatment or any abnormal clinical findings that required medical referral.

**Table 1:** Participant Demographics

	N	Age	Height	BMI	Body Weight	Fat-Free Mass	Body Fat
		(y)	(cm)	(kg/m^2^)	(kg)	(kg)	(%)
Overall	25	21.7 ± 3.0	176.1 ± 8.2	25.0 ± 3.0	78.2 ± 13.0	61.0 ± 11.6	15.2 ± 5.2
Male	20	21.4 ± 2.7	178.9 ± 5.9	25.9 ± 2.4	83.0 ± 8.5	65.5 ± 7.2	14.0 ± 4.8
Female	5	23.2 ± 3.1	164.8 ± 5.4	21.7 ± 2.4	59.3 ± 8.5	42.9 ± 5.9	20.1 ± 3.2

Data are means ± standard deviations

### Cardiovascular markers

Table 2 shows HR, systolic blood pressure (SBP), and diastolic blood pressure (DBP) responses observed among treatments. No significant treatment x time interactions were observed in HR, SBP, or DBP responses obtained prior to and during the REE test. Additionally, no noticeable changes were observed in resting 12-lead ECG recordings.

**Table 2:** Heart Rate and Blood Pressure Response

Variable	Treatment	Time (min)					p-level
		Pre	0	10	20	30	
HR (beats/min)	PLA	58.2 ± 9.5	60.4 ± 8.1	58.6 ± 8.5	59.6 ± 8.3	60.6 ± 8.4	0.84
	PWS	55.5 ± 7.3	58.2 ± 9.5	54.2 ± 7.2	56.5 ± 7.7	58.1 ± 8.1	
	PWS + S	56.6 ± 7.2	57.5 ± 8.8	56.5 ± 9.5	58.4 ± 9.1	60.4 ± 10.8	
SBP (mmHg)	PLA	113.3 ± 7.6	114.3 ± 8.0	112.7 ± 6.8	114.3 ± 6.3	113.6 ± 6.6	0.52
	PWS	111.5 ± 7.1	112.4 ± 7.3	112.9 ± 8.6	112.8 ± 7.9	112.5 ± 8.2	
	PWS + S	113.1 ± 7.8	112.5 ± 8.3	113.0 ± 9.6	113.9 ± 10.6	114.6 ± 10.0	
DBP (mmHg)	PLA	69.6 ± 6.8	69.2 ± 6.5	68.8 ± 6.3	69.5 ± 7.4	69.1 ± 7.1	0.51
	PWS	69.0 ± 6.7	70.7 ± 6.1	70.9 ± 7.1	70.6 ± 7.5	70.4 ± 7.7	
	PWS + S	69.6 ± 7.2	70.0 ± 6.7	71.0 ± 6.8	71.1 ± 8.1	68.4 ± 13.0	

Data are means ± standard deviations for Heart Rate (HR), Systolic Blood Pressure (SBP), and Diastolic Blood Pressure (DBP). MANOVA analysis revealed overall Wilks’ Lambda treatment (*p* = 0.28), time (*p* = 0.03), and treatment x time (*p* = 0.81). Greenhouse-Geisser univariate p-levels of interactions (treatment x time) are reported above

### Resting energy expenditure

**Fig. 3:**
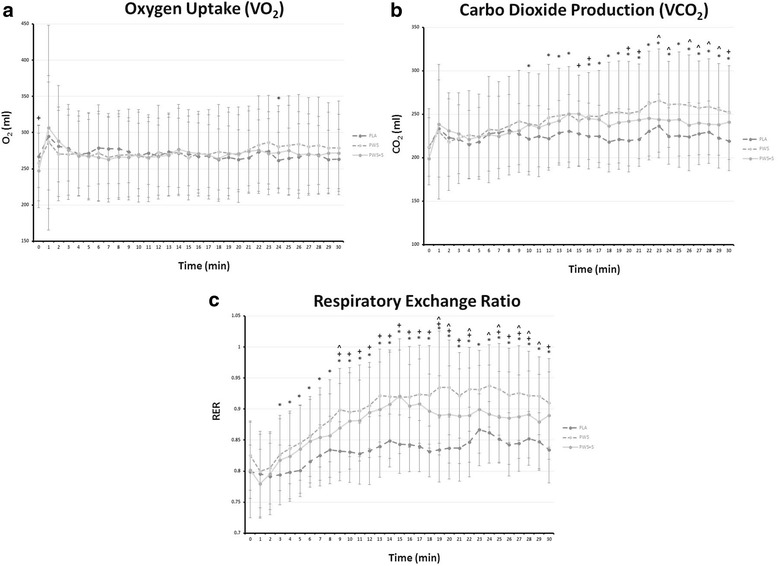
Oxygen update (Panel **a**), carbon dioxide production (Panel **b**), and respiratory exchange ratio values (Panel **c**) observed during the first 30-min following supplementation. Data are mean ± SD. ^ represents *p* < 0.05 difference between PLA and PWS; + represents *p* < 0.05 difference between PLA and PWS + S; and, * represents *p* < 0.05 difference between PWS and PWS + S

Figure 3 presents oxygen uptake (VO_2_), carbon dioxide production (VCO_2_), and respiratory exchange ratio (RER) values (VCO_2_/VO_2_) observed during the post-supplementation REE test. MANOVA revealed an overall significant Wilks’ Lambda treatments x time effect (*p* < 0.001) as well as significant univariate ANOVA treatments x time interactions VCO_2_ (*p* = 0.008) and RER values (*p* = 0.001). Post-hoc analysis revealed that PWS and PWS + S ingestion primarily effected VCO_2_ and RER responses compared to the PLA treatment and that PWS ingestion promoted higher VCO_2_ and RER values toward the end of the 30 min REE test. Analysis of AUC changes from baseline revealed significant differences among treatments in VO_2_ (PLA 684 ± 376; PWS 802 ± 434; PWS + S 1,034 ± 584 ml/min, *p* = 0.015), VCO_2_ (PLA 634 ± 262; PWS 1,151 ± 673; PWS + S 1,372 ± 604 ml/min, *p* < 0.001), and RER (PLA 1.48 ± 0.67; PWS 2.44 ± 0.98; PWS + S 2.79 ± 0.89, *p* < 0.001). Post-hoc analysis revealed that VO_2_ AUC in the PWS + S treatment was significantly greater than the PLA treatment (*p* = 0.013, CI [64, 636]) but not the PWS treatment. VCO_2_ and RER AUC values were significantly higher in the PWS (VCO_2_ *p* < 0.001, CI [242, 792]; RER *p* < 0.001, CI [−.43, −1.53]) and PWS + S (VCO_2_ *p* < 0.001, CI [−1,036, −440]; RER *p* < 0.001, CI [−1.87, −0.78]) compared to the PLA treatment. However, no significant differences were observed between PWS and PWS + S treatments.

### Perceptions of readiness to perform

Perceptions about readiness to perform are presented in Table 3. MANOVA revealed a significant overall Wilks’ Lambda treatments x time interaction (*p* = 0.004) in perceptions of readiness to perform questions. Significant univariate ANOVA treatments x time effects were also observed in response to “*I am optimistic about my future performance*” and “*I feel vigorous and energetic*”. Post-hoc analysis revealed that participants in the PWS and PWS + S treatments rated optimism about performance and feelings of vigor and energy higher than the PLA treatment after 1 h of ingestion. However, perceptions about optimism about performance and vigor and energy in the PWS + S treatment were lower than PLA and PWS responses after 2 h. No significant treatments x time interactions were seen in remaining questions.

**Table 3:** Readiness to Perform Visual Analogue Scale

Questions	Treatment	Time (h)			p-level
		Pre	1	2	
I am looking forward to today’s workout	PLA	3.67 ± 0.85	3.61 ± 1.00	3.61 ± 0.87	0.21
	PWS	3.71 ± 0.77	3.89 ± 0.79	3.65 ± 0.65	
	PWS + S	3.71 ± 0.99	3.82 ± 1.17	3.51 ± 1.09	
I am optimistic about my future performance	PLA	3.74 ± 0.83	3.70 ± 0.94	3.78 ± 0.88	0.02_q_
	PWS	3.88 ± 0.69	4.05 ± 0.73 ^a^	3.83 ± 0.70	
	PWS + S	4.00 ± 0.69	4.21 ± 0.62 ^a^	4.01 ± 0.71	
I feel vigorous and energetic	PLA	3.23 ± 0.95	3.35 ± 0.90	3.38 ± 1.04	0.001
	PWS	3.19 ± 0.89	3.77 ± 0.78 *^a^	3.33 ± 1.09	
	PWS + S	3.33 ± 0.77	3.89 ± 0.73 *^a^	2.96 ± 0.97 ^a,b^	
I have little muscle soreness	PLA	3.60 ± 0.99	3.42 ± 0.99	3.29 ± 1.01	0.17
	PWS	3.54 ± 1.17	3.81 ± 1.35	3.25 ± 1.03	
	PWS + S	3.13 ± 1.31	3.27 ± 1.28	3.21 ± 1.12	

Values are means ± standard deviations. MANOVA analysis revealed overall Wilks’ Lambda treatment (*p* = 0.03), time (*p* = 0.01), and treatment x time (*p* = 0.004). Greenhouse-Geisser linear or quadratic (_q_) univariate p-levels of interactions (treatment x time) are reported above * represents *p* < 0.05 difference from baseline. ^a^ denotes a significant difference from PLA. ^b^ denotes a significant difference from PWS. ^c^ denotes a significant difference from PWS + S

### Cognitive function

**Fig. 4:**
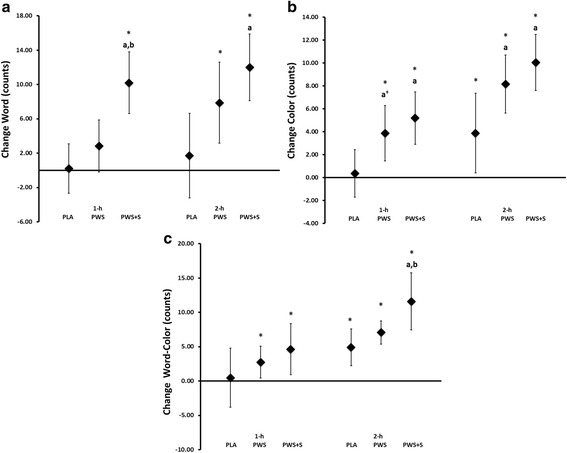
Changes in Stroop Word (Panel **a**), Color (Panel **b**), and Word-Color (Panel **c**) counts. Data are mean change and 95% CI. * represents *p* < 0.05 difference from baseline, a = *p* < 0.05 from PLA, b = *p* < 0.05 from PWS, c = *p* < 0.05 difference from PWS + S. ^†^ represents *p* > 0.05 to *p* < 0.10 difference

Table 4 presents Stroop Color-Word cognitive function results. An overall Wilk’s Lambda treatments x time interaction effect was observed among treatments (*p* < 0.001). Significant univariate ANOVA treatment x time interactions were also observed in Word, Color and Word-Color scores. Post-hoc analysis revealed that Word, Color and Word-Color scores significantly increased over time in the PWS and/or PWS + S treatments with values in the PWS and PWS + S treatments significantly greater than the PLA at several data points. Figure 4 presents changes from baseline with 95% CI values. These graphs show that changes in Word, Color, and Word-Color were significantly increased above baseline (i.e., mean and 95% CI above baseline) more consistently in the PWS and/or PWS + S treatments compared to the PLA treatment. Additionally, changes in the PWS and/or PWS + S were significantly greater than PLA responses at several data points. There was also some evidence that changes in the PWS + S treatment were significantly greater than the PWS treatment at several data points. However, it should be noted that pre-supplementation values in the PWS + S treatment were lower at baseline compared to PLA and PWS responses so these data should be interpreted with caution.

**Table 4:** Stroop Word-Color Test

Variable	Treatment	Time (h)			p-level
		Pre	1	2	
Word (counts)	PLA	120.9 ± 15.7	121.1 ± 16.6	122.6 ± 18.0	<0.001
	PWS	116.7 ± 15.5 ^a^	119.5 ± 15.4	124.6 ± 17.7 *	
	PWS + S	104.8 ± 11.4 ^a,b^	115.0 ± 13.6 *^a,c^	116.8 ± 14.4 *^a,b^	
Color (counts)	PLA	89.9 ± 12.4	90.2 ± 13.6	93.8 ± 17.1	0.02
	PWS	91.2 ± 21.3	95.1 ± 19.9	99.4 ± 21.0 *^c^	
	PWS + S	79.7 ± 10.5 ^a,b^	84.9 ± 11.7 ^a,b^	89.8 ± 12.5 *^b^	
Word-Color (counts)	PLA	66.4 ± 9.6	66.8 ± 14.9	71.3 ± 11.7	0.037
	PWS	67.5 ± 20.3	70.2 ± 21.6	74.6 ± 18.9 *	
	PWS + S	52.4 ± 10.6 ^a,b^	57.0 ± 10.4 ^a,b^	64.0 ± 10.8 *^a,b^	

Values are means ± standard deviations for Stroop Word, Color, and Word-Color tests. MANOVA analysis revealed overall Wilks’ Lambda treatment (*p* < 0.001), time (*p* < 0.001), and treatment x time (*p* < 0.001). Greenhouse-Geisser univariate p-levels of interaction (treatment x time) are reported above. * represents *p* < 0.05 difference from baseline. ^a^ denotes a significant difference from PLA. ^b^ denotes a significant difference from PWS. ^c^ denotes a significant difference from PWS + S

### Performance assessment

Table 5 shows bench press and leg press lifting volume observed during the 3rd set to failure and for all three sets of exercise while Table 6 presents Wingate anaerobic sprint capacity results. All participants completed 10 repetitions during the first set. One of participants was only able to complete 7 repetitions during the second set but this was consisted in each trial. MANOVA revealed tendencies toward significant differences among treatments in bench press 3rd set lifting volume (*p* = 0.086, partial η^2^ = 0.08) and total lifting volume (*p* = 0.10, partial η^2^ = 0.08). Lifting volume for the 3rd set to failure also tended to be greater in the PWS + S compared to the PLA treatment (*p* = 0.057, partial η^2^ = 0.09, Cohen’s D = 0.18). No significant differences were observed in leg press or cycling sprint peak power, mean power, or total work.

**Table 5:** Bench Press and Leg Press Endurance

Variable	Treatment	Total Volume	p-level
Bench Press 3rd Set Lifting Volume (kg)	PLA	879 ± 440	0.086
	PWS	933 ± 460	
	PWS + S	962 ± 500 ^a^	
Leg Press 3rd Set Lifting Volume (kg)	PLA	6,712 ± 3,538	0.29
	PWS	7,295 ± 3,589	
	PWS + S	6,825 ± 3016	
Bench Press Total Lifting Volume (kg)	PLA	2,166 ± 718	0.10
	PWS	2,217 ± 732	
	PWS + S	2,268 ± 809	
Leg Press Total Lifting Volume (kg)	PLA	12,550 ± 4,321	0.30
	PWS	13,139 ± 4,409	
	PWS + S	12,701 ± 3,866	

Values are means ± standard deviations. MANOVA analysis revealed overall Wilks’ Lambda of *p* = 0.098 for 3rd set lifting volume and *p* = 0.117 for total lifting volume. Greenhouse-Geisser univariate p-levels are presented above. ^a^ represents *p* < 0.057 difference from PLA. ^c^ represents *p* < 0.057 difference from PWS + S

**Table 6:** Wingate Anaerobic Capacity Test

Variable	Treatment	Value	p-level
Peak Power (Watt)	PLA	1,578 ± 510	0.46
	PWS	1,502 ± 561	
	PWS + S	1,491 ± 515	
Mean Power (Watt)	PLA	602 ± 131	0.61
	PWS	595 ± 145	
	PWS + S	582 ± 188	
Peak Power (Watt/kg)	PLA	19.8 ± 5.1	0.51
	PWS	19.0 ± 5.2	
	PWS + S	18.8 ± 5.2	
Mean Power (Watt/kg)	PLA	7.6 ± 1.0	0.78
	PWS	7.6 ± 1.2	
	PWS + S	7.7 ± 1.3	
Total Work (Joules)	PLA	17,662 ± 4,605	0.49
	PWS	17,850 ± 4,340	
	PWS + S	18,203 ± 4,658	

Values are means ± standard deviations. MANOVA analysis revealed overall Wilks’ Lambda group (*p* = 0.89). Greenhouse-Geisser univariate p-levels are reported above

### Blood chemistry

Table 7 presents plasma creatine values observed in response to supplementation treatments. Results revealed that PWS and PWS + S ingestion significantly increased plasma creatine levels after 1 h and 2 h in comparison to the PLA treatment. Tables 8 and 9 present pre- and 2 h post supplementation/exercise blood chemistry panels. No significant interactions were observed among treatments in ALP, ALT, AST, CK, or LDH. Significant interactions were observed among treatments in BUN, creatinine, and BUN to creatinine ratio. As expected, PWS and PWS + S supplementation (which contained about 1.3 g of creatine) resulted in a small increase in creatinine in which values were well-within normal limits for active individuals [53, 54]. BUN and the BUN to creatinine ratio decreased indicative of less whole body catabolism. No significant interactions were observed among treatments in HDL-C, LDL-C or triglyceride levels. Significant treatments x time effects were observed in total cholesterol, the ratio of CHOL: HDL and blood glucose levels. However, given that participants ingested caffeine with a small amount of maltodextrin prior to exercise, these changes were expected and within normal expected values for trained individuals undergoing intense exercise [53–56] as well as within normal clinical ranges [57].

**Table 7:** Plasma Creatine

Variable	Treatment	Time (h)			p-level
		0	1	2	
Plasma Creatine	PLA	0.74 ± 0.27	0.69 ± 0.21	0.71 ± 0.24	<0.001
(μmol/L)	PWS	0.71 ± 0.19	3.13 ± 1.43 *^a^	3.10 ± 1.45 *^a^	
	PWS + S	0.68 ± 0.17	3.28 ± 1.13 *^a^	3.89 ± 1.61 *^a,b^	

Values are means ± standard deviations. MANOVA analysis revealed Wilks’ Lambda treatment (*p* < 0.001), time (*p* < 0.001) and treatment x time effects (*p* < 0.001). The Greenhouse-Geisser univariate treatment x time interaction is reported above. * represents *p* < 0.05 difference from baseline. ^a^ denotes a significant difference from PLA. ^b^ denotes a significant difference from PWS. ^c^ denotes a significant difference from PWS + S

**Table 8:** Muscle and Liver enzymes and Markers of Catabolism

Variable	Treatment	Time (h)		p-level
		0	2	
ALP	PLA	79.8 ± 21.7	85.7 ± 27.5	0.70
(U/L)	PWS	77.7 ± 31.1	82.3 ± 22.5	
	PWS + S	75.0 ± 19.2	82.2 ± 23.0	
ALT	PLA	29.5 ± 27.6	33.3 ± 32.9	0.26
(U/L)	PWS	24.5 ± 11.0	28.0 ± 12.2	
	PWS + S	25.7 ± 11.7	27.1 ± 10.1	
AST	PLA	38.1 ± 60.6	44.9 ± 75.7	0.37
(U/L)	PWS	24.9 ± 6.2	29.6 ± 7.1	
	PWS + S	25.3 ± 6.6	28.8 ± 6.5	
CK	PLA	713 ± 2354	836 ± 2786	0.33
(U/L)	PWS	191 ± 104	231 ± 111	
	PWS + S	189 ± 85	223 ± 85	
LDH	PLA	182 ± 77	205 ± 115	0.55
(U/L)	PWS	161 ± 18	187 ± 22	
	PWS + S	167 ± 26	192 ± 24	
BUN	PLA	15.9 ± 4.8	14.4 ± 4.1	0.013
(mg/dl)	PWS	17.4 ± 4.7	15.1 ± 3.7*	
	PWS + S	16.4 ± 4.0	15.9 ± 3.5	
Creatinine	PLA	0.99 ± 0.14	1.05 ± 0.16	0.037
(mg/dl)	PWS	1.09 ± 0.17 ^a,c^	1.23 ± 0.17 *^a^	
	PWS + S	0.97 ± 0.14	1.11 ± 0.17 *^a,b^	
BUN: Creatinine	PLA	16.2 ± 5.9	13.9 ± 4.9 *	<0.001
	PWS	16.2 ± 5.0	12.3 ± 3.2 *^a,c^	
	PWS + S	17.1 ± 4.8	14.4 ± 3.4 *	

Values are means ± standard deviations for alkaline phosphatase (ALP), aspartate transaminase (AST), alanine transaminase (ALT), creatine kinase (CK), lactate dehydrogenase (LDH), blood urea nitrogen (BUN), and creatinine. MANOVA analysis revealed overall Wilks’ Lambda treatment (*p* < 0.001), time (*p* < 0.001), and treatment x time (*p* < 0.001) effects. Greenhouse-Geisser univariate p-levels of interaction (treatment x time) are reported above. * represents *p* < 0.05 difference from baseline. ^a^ denotes a significant difference from PLA. ^b^ denotes a significant difference from PWS. ^c^ denotes a significant difference from PWS + S

**Table 9:** Blood Lipid and Glucose Panel

Variable	Treatment	Time (h)		p-level
		0	2	
Cholesterol	PLA	170.6 ± 34.6	182.8 ± 35.1	0.006
(mg/dl)	PWS	187.3 ± 46.6 ^a^	180.2 ± 36.9	
	PWS + S	161.7 ± 29.8 ^a,b^	174.0 ± 33.5 ^a^	
HDL-c	PLA	54.9 ± 13.3	60.1 ± 16.0	0.57
(mg/dl)	PWS	52.2 ± 13.9	58.9 ± 14.6	
	PWS + S	54.2 ± 16.2	60.1 ± 18.1	
CHOL: HDL-c	PLA	3.2 ± 0.9 ^b^	3.2 ± 0.9	0.001
	PWS	3.8 ± 1.3 ^a,c^	3.2 ± 1.0 *	
	PWS + S	3.2 ± 1.0 ^b^	3.1 ± 1.0	
LDL-c	PLA	104.3 ± 36.7	109.6 ± 41.2	0.55
(mg/dl)	PWS	96.9 ± 31.9	104.5 ± 36.0	
	PWS + S	99.8 ± 41.2	104.6 ± 40.7	
Triglycerides	PLA	98.0 ± 58.6	89.3 ± 38.3	0.14
(mg/dl)	PWS	109.5 ± 56.8	100.3 ± 47.5	
	PWS + S	88.3 ± 42.2	92.8 ± 54.6	
Glucose	PLA	90.2 ± 9.7	101.5 ± 14.4 *	0.03
(mg/dl)	PWS	100.1 ± 16.0 ^a,c^	114.4 ± 12.6 *^a^	
	PWS + S	90.4 ± 7.3	113.8 ± 15.0 *^a^	

Values are means ± standard deviations for total cholesterol (CHOL), high density lipoproteins (HDL-c), the CHOL: HDL ratio (CHOL: HDL-c), low density lipoproteins (LDL-c), triglycerides and blood glucose. MANOVA analysis revealed overall Wilks’ Lambda treatment (*p* < 0.001), time (*p* < 0.001), and treatment x time (*p* < 0.001). Greenhouse-Geisser univariate p-levels of interaction (treatment x time) are reported above. * represents *p* < 0.05 difference from baseline. ^a^ denotes a significant difference from PLA. ^b^ denotes a significant difference from PWS. ^c^ denotes a significant difference from PWS + S

## Discussion

The purpose of this study was to examine the effects of acute ingestion of a PWS with and without *p*-synephrine on resting energy expenditure, perceptions of readiness to perform, cognitive function, and anaerobic exercise performance. Additionally, to examine the safety of acute ingestion of these PWS’s on resting heart rate, blood pressure, 12-lead ECG tracings, and standard clinical blood chemistry panels. Results revealed that ingestion of these PWS’s did not promote clinically significant changes in heart rate, blood pressure, ECG findings, or clinical blood markers. Thus, acute ingestion of these PWS’s prior to exercise appeared to be well-tolerated when consumed by young, healthy individuals. The primary effect of ingesting these PWS’s prior to exercise appeared to be an increase resting energy expenditure, improved perceptions about readiness to perform, and improved cognitive function. There were limited to no effects on bench press and leg press muscular endurance as well as anaerobic sprint capacity. Moreover, adding 20 mg of *p*-synephrine to the PWS provided little to no additive benefits. The following sections provide a more detailed analysis of results observed and comparison to available literature.

### Resting energy expenditure

A number of studies have reported that ingestion of energy drinks and/or thermogenic type supplements primarily containing caffeine can increase resting metabolism [58–65]. For example, Taylor and colleagues [65] reported that ingestion of caffeine enriched coffee (400 mg) increased REE by approximately 14% for up to 3-h compared to consumption of coffee containing standard amounts of caffeine (200 mg). Rudelle and coworkers [66] reported that consuming beverages containing green tea catechins, caffeine, and calcium for 3-days increased energy expenditure by 106 ± 31 kcal/24 h. Ryan et al. [62] reported that ingestion of a thermogenic supplement containing caffeine, capsaicin, bioperine, and niacin increased energy expenditure and oxygen update values. Wilborn and associates [64] reported that ingestion of a thermogenic supplement containing green tea extract and caffeine increased REE by 15-19% compared to 0.3–2.5% when ingesting a placebo. Campbell and coworkers [60] reported that ingestion of a supplement containing 200 mg of caffeine and green tea extract significantly increased resting metabolic rate from 7 to 9% for up to 3 h after ingestion. Moreover, Miles-Chan and colleagues [67] reported that consumption of a sugar free energy drink containing 120 mg of caffeine increased REE by about 4% and that the changes observed were due to the caffeine in the drinks rather than other nutrients (i.e., taurine and glucuronolactone).

*Citrus aurantium* (generally containing 20–100 mg of *p*-synephrine) has also been purported to increase resting energy expenditure and/or effect carbohydrate and fat oxidation rates [30–33]. For example, Ratamass and colleagues [31] reported that ingestion of 100 mg of *p*-synephrine increased lipolysis primarily at rest as well as post-exercise oxygen uptake, energy expenditure, and fat oxidation. Sale et al. [33] reported that ingesting a supplement containing 6 mg of *p-*synephrine, 150 mg caffeine, and 150 mg catechin polyphenols increased resting and exercise carbohydrate oxidation rates. Additionally, Gutiérrz-Hellín and Del Coso [32] reported that acute *p*-synephrine ingestion (3 mg/kg) increased fat oxidation rates while exercising at low-to-moderate exercise intensities. Thus, there is support to the rationale that adding *p*-synephrine to a PWS can increase metabolism and affect substrate utilization.

Results of the present study provide some support to this contention. In this regard, mean oxygen uptake during the 30 min REE test was increased by about 1.4% in the PLA treatment compared to 5.6% in the PWS treatment and 8.6% in the PWS + S treatment. There was also evidence that PWS and PWS + S ingestion significantly increased resting VCO_2_ and RER to a greater degree than in the PLA treatment indicating greater carbohydrate oxidation. Analysis of AUC changes from baseline indicated that while PWS had a greater impact on VO_2_ VCO_2_ and RER values; the PWS + S observed greater changes from baseline compared to the PLA treatment. These findings are consistent with several reports indicating that ingestion of thermogenic type supplements increases energy metabolism and/or carbohydrate oxidation [33, 62, 66, 67] while contrasting other studies reporting either no effects [64] or greater fat oxidation [32]. However, ingestion of PWS + S treatment did not result in higher mean VO_2_ values than the PWS treatment. Therefore, the addition of *p*-synephrine to the PWS did not promote greater overall energy expenditure. Whether adding higher levels of *p-*synephrine would promote added benefits remains to be determined.

### Perceptions of readiness to perform and cognitive function

Numerous studies indicate that ingesting caffeine containing energy drinks or supplements can improve mental focus and/or cognition [1, 4, 68–70]. There is also evidence that *Citrus aurantium* ingestion can affect memory [40, 41]. Therefore, it’s plausible that ingestion of a PWS containing caffeine and/or *p*-synephrine could affect mental focus and/or cognitive function prior to and/or during exercise. In the present study, participants ingesting the PWS’s indicated they felt more optimistic about performance and more vigorous and energetic after ingestion the PWS’s compared to the PLA treatment. Additionally, there was evidence that ingestion of the PWS and PWS + S consistently increased Word, Color, and Word-Color scores from baseline and that some of these changes were greater than PLA responses after 1 or 2 h. However, it should be noted that participants started the PWS + S treatment with lower scores prior to supplementation and the scores in the PWS + S treatment did not exceed PLA or PWS responses at any data point so these results should be interpreted with caution. Further, the addition of 20 mg of *p*-synephrine to the PWS supplement used in our study did not appear to provide additive benefit. Results are in agreement with Hoffman et al. [71] who reported a significantly greater feeling of energy and focus compared to placebo after ingesting a supplement containing a PWS but contrast those of Gonzales and associates [72] who reported no significant difference in VAS ratings of energy when ingesting a supplement containing caffeine, creatine, β-alanine. However, the participants in that study were administered the supplement 10-min before performing resistance-exercise which may have limited results since it takes about an hour for caffeine levels in the blood to peak after caffeine ingestion [1, 4].

### Exercise performance

Numerous studies have evaluated the effects of ingesting PWS’s and/or energy drinks containing a variety of nutrients on exercise performance [1]. For example, Walsh and colleagues [70] reported that ingestion of a PWS containing 2.05 g of an energy matrix (caffeine, taurine, glucuronolactone) with amino acids significantly improved perceptions of energy, decreased perceptions of fatigue, an improved run time to exhaustion at 70% of VCO_2_ max. Gonzalez et al. [72] reported that acute ingestion of a pre-workout supplement containing caffeine, β-alanine, and creatine significantly increased the number of repetitions performed at 80% of 1RM as well as peak and mean power of bench press. Additionally, Ratamess et al. [30] reported that acute supplementation of PWS’s containing *p*-synephrine (100 mg), p-synephrine (100 mg) and caffeine (100 mg) increased the number of repetitions and lifting volume (6 sets of squats for up to 10 repetitions at 80% 1RM) compared to control and placebo treatments. Differences were more noted in the final 3 sets of exercise. Further, mean power and lifting velocity was reported to be higher for the caffeine and *p*-synephrine treatment compared to control and placebo treatments. Consequently, there is evidence that ingestion of caffeine and/or p-synephrine prior to exercise may affect high intensity exercise performance.

In the present study, no clear ergogenic benefit of PWS or PWS + S ingestion was observed. While participants in the PWS + S treatment tended to perform more total work during the final set of bench press, no overall differences were observed in bench press or leg press lifting volume. Additionally, no differences were observed among treatments in the Wingate Anaerobic Capacity cycling sprint test. Therefore, while participants reported they had more energy and were more optimistic about performance, it did not result in statistically significant differences in upper and lower body muscular endurance or sprint capacity. Whether adding more *p*-synephrine to the PWS or regular use of these PWS’s during training may yield more definitive ergogenic benefit remains to be unclear.

### Safety

One of the criticisms of use of energy drinks and/or PWS’s containing caffeine and/or *p*-synephrine is that they may increase heart, blood pressure, and/or prevalence of arrhythmias [1, 73, 74]. In the present study, acute ingestion of PWS or PWS + S did not significantly increased heart rate, blood pressure, or effect resting ECG’s. Additionally, although there were some expected changes in blood markers in response to exercise (hemoconcentration) and/or ingestion of maltodextrin, creatine, and caffeine containing PWS’s (i.e., evidence of increased creatinine, lipolysis, and blood glucose release); values remained within normal expected values for trained individuals undergoing intense exercise [53–56] as well as within normal clinical ranges [57]. In this regard, creatinine levels expectedly increased to a greater degree in the PWS and PWS + S but these values were small and well-within normal ranges for active individuals particularly when taking creatine [2, 6, 25, 53]. Moreover, while significant interactions were observed among treatments in BUN and the ratio of BUN: Creatinine, all values decreased suggesting less general whole body catabolism. Additionally, while significant differences were seen in total cholesterol among treatments, total cholesterol did not significantly change from baseline in any treatment and the CHOL: HDL-c ratio either was unchanged or decreased suggesting reduced risk. Finally, while differences were observed among treatments in pre- and post-exercise/supplementation blood glucose values and blood glucose was higher with PWS ingestion suggesting greater hepatic glucose release (which would be beneficial during exercise); no significant differences were observed between PWS and PWS + S treatments and all treatments and were within normal expected exercise ranges [57]. We also did not observe any adverse events related to the study that required medical treatment or clinical findings that required medical referral. Consequently, ingestion of these PWS’s prior to exercise did not appear to promote any clinically relevant changes in these markers. These findings support prior reports that ingestion of PWS’s and/or energy drinks do not appear to pose undo health risk in apparently healthy individuals [58, 59, 63, 75–78].

## Conclusion

Within the limits of the present study, acute ingestion of PWS and/or PWS + S prior to exercise appears to be well-tolerated when consumed by young, healthy individuals. The primary effects appear to be to increase resting energy expenditure responses and improve perceptions about readiness to perform and cognitive function with limited to no effects on muscular endurance and no effects on anaerobic sprint capacity. The addition of 20 mg of *p*-synephrine to the PWS yielded limited to no additive benefits. Whether regular use of the PWS’s used in this study and/or higher amounts of *p*-synephrine may promote greater benefits remains to be determined. Given the widespread use of PWS’s and energy drinks, additional study to examine the safety and efficacy is warranted.
